# Morphometric description of the koala humerus using microcomputed tomography

**DOI:** 10.1038/s41598-022-22944-0

**Published:** 2022-10-27

**Authors:** Jason Hawkins, Rachel M. Basa, Matthew J. Norton, Kenneth A. Johnson

**Affiliations:** Faculty of Science, Sydney School of Veterinary Science, University of Sydney, Camperdown, NSW 2006 Australia

**Keywords:** Anatomy, Musculoskeletal system

## Abstract

The current prognosis for successful return to function in koalas with appendicular fractures is poor despite being the most common fracture type to result in successful rehabilitation. The forelimb, particularly the humerus, plays a critical role in stabilisation and support while climbing trees. Successful rehabilitation therefore requires adequate internal stabilisation to promote bone healing and faster return to function. Current knowledge of koala limb bone morphometry is lacking and would provide useful clinical insight for future orthopaedic research, particularly with regards to recommendations regarding implant size and type. In this study microcomputed tomography (micro-CT) was used to describe bone length, internal and external diameters, and cortical thickness at five transverse levels along the humerus of skeletally mature koala cadavers. Qualitative descriptions were also made regarding bone features deemed clinically relevant to potential fracture repair techniques. Mean humeral length was 114.3 mm (95% CI 107.29–121.31 mm). Mediolateral diameters were greater than craniocaudal diameters at each measurement level, and the diaphysis has a distally tapering medullary cavity. Diaphyseal cortices were relatively homogenous with slight distal thickening, and medial cortices were thickest along the entire bone. The bone protuberances of the deltoid and supinator ridges projected most of the way down the lateral surface of the bone while the medial surface remained relatively uniform. Distal to the deltoid ridge the humerus curved caudally, terminating at a craniocaudally flattened distal epiphysis. Morphometric descriptions provided in this study will serve as a useful reference for future research, guiding orthopaedic surgery and improving prognosis of koala humeral fractures.

## Introduction

Traumatic injuries are the most common cause of wild koala admissions to wildlife hospitals^1^. Prognosis is generally poor, with appendicular fracture injuries being the most common type to result in successful rehabilitation and release^2^. Despite this, less than 10% of appendicular fracture cases end in successful release^2^. Euthanasia is more often recommended with forelimb than hindlimb injuries as koalas are highly dependent on their forelimb for climbing stability and support^2^. The humerus in turn plays a critical role as the attachment site of many muscles involved in maintaining climbing stability. Despite concurrent reasons for euthanasia and poor availability of veterinary resources^3^ efforts to rehabilitate through orthopaedic repair do occur and should therefore be optimised to provide better welfare outcomes.

Morphometry is the quantitative study of external shape and dimensions. Current literature detailing koala appendicular skeletal morphometry does not exist. Anatomical studies on koalas have historically focussed on gross descriptions of koala bones and muscular anatomy^4–8^. More recent publications have provided morphometric descriptions of the koala skull and nasal cavity^9,10^. Morphometric and biomechanical studies have been carried out using feline^11^ canine^12–15^, production animal^16–18^, and human^19,20^ bones to provide useful clinical insights for further orthopaedic research. These descriptive studies help develop standardised surgical approach techniques and recommendations regarding orthopaedic implant size and type. In koalas, prognosis for humeral injuries may be improved using precisely engineered fixation techniques to promote optimal healing and faster return to function.

Fracture treatment techniques aim to provide long-lasting biomechanical stability^21^, preserve articular structures, spare blood supply and promote healing^22,23^. Implant failures are largely attributed to implant size and design^24^. Therefore, orthopaedic research must draw from anatomical studies to develop implants of different types and sizes best indicated for specific fracture repairs.

This study aims to describe morphometric parameters of the koala humerus, including bone length, cortical thickness, and internal and external bone diameters at various predetermined locations along the humerus of skeletally mature koalas using microcomputed tomography (micro-CT). It also aims to qualitatively describe any unique or interesting bone features deemed to be clinically relevant to fracture repair by internal fixation with implants. It is anticipated that understanding koala humeral bone morphometry will facilitate a more ideal surgical approach to stabilise fractures and improve the prognosis of injury. Additionally, this study aims to discuss whether the data obtained could help guide future orthopaedic research in recommending surgical implant parameters best indicated for koala humeral fracture repair.

## Results

### Bone length and descriptive features

Koala humeral length was a mean of 114.3 mm (95% CI 107.29–121.31 mm).

**Figure 1:**
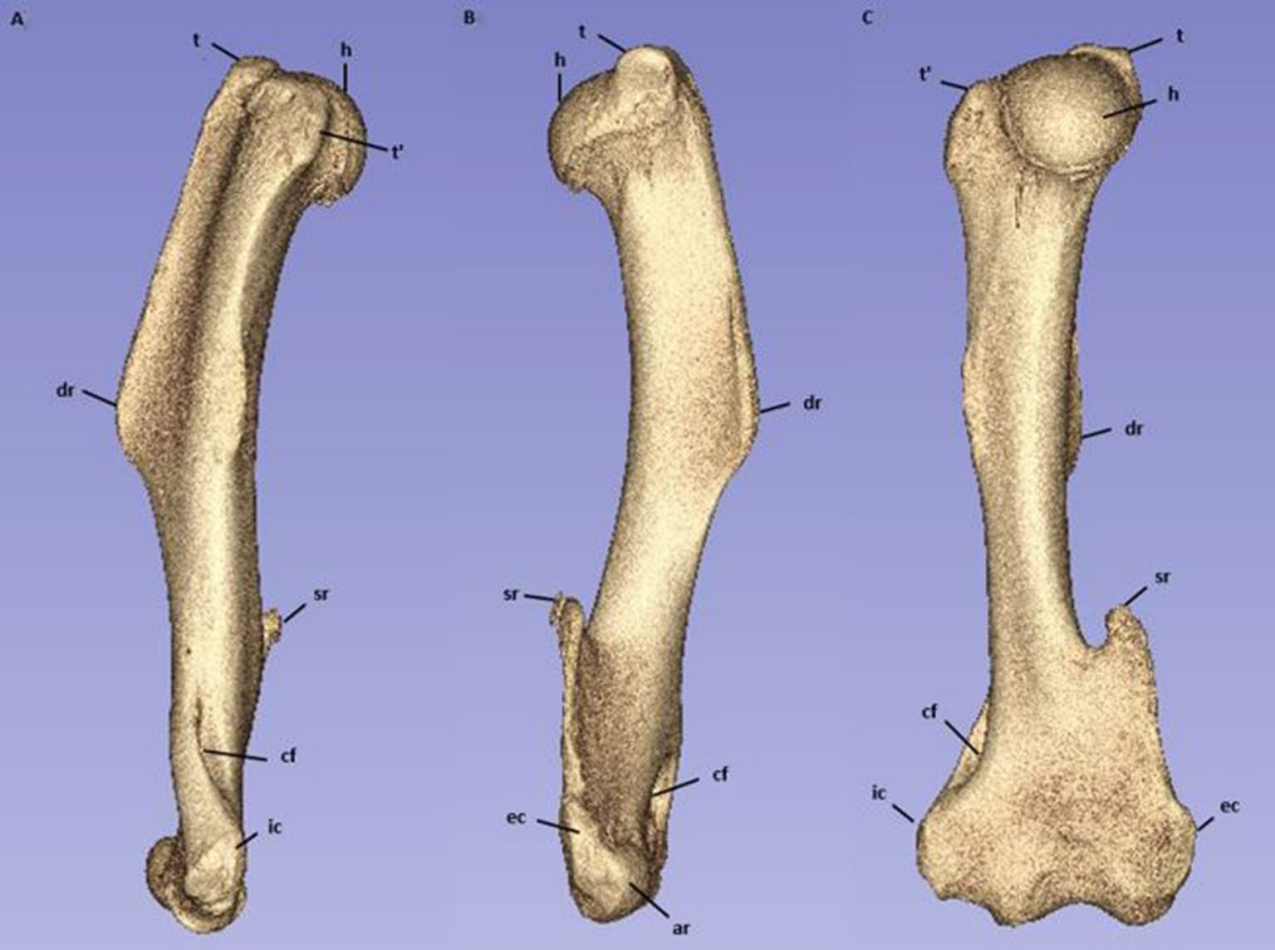
3D reconstruction of the koala humerus from micro-CT scan using Slicer version 4.10.2. Inluding (**A**) medial view with left of the image being cranial, (**B**) lateral view with the right of the image being cranial, and (**C**) Caudal view with left of the image being medial. Identifying landmarks are also identified. Where h = humeral head; t = greater tuberosity; t’ = lesser tuberosity; dr = deltoid ridge; sr = supinator ridge; cf = supracondylar foramen; ec = external epicondyle; ic = internal epicondyle; ar = articular surface (radius); au = articular surface (ulna).

The greater and lesser tuberosities were highly prominent. In four of six samples the greater tuberosity protruded proximally, past the most proximal aspect of the humeral head. The deltoid ridge was very pronounced, coursing distally on the craniolateral surface of the bone from the greater tuberosity, attenuating before the distal third measurement level. The deltoid ridge measurement level was on average 48.12 mm (95% CI 44.66–51.58 mm), or 42% the length from the most proximal point of the humeral head. After the deltoid ridge disappeared distally the bone curved caudally (Fig. 1). There was also a highly pronounced supinator ridge on the lateral surface just distal to the distal third measurement level. In the region of the distal epiphysis, the humerus flattened craniocaudally and flared in the mediolateral plane. Both medial and lateral epicondyles were moderately pronounced.

### External diameter of bone

Humeral external diameters were reported at all five measurement levels (Table 1). Along the entire length of the bone mediolateral external diameter was on average greater than craniocaudal diameter. The widest craniocaudal diameter was between tuberosities, while the widest mediolateral diameter was between the epicondyles at the proximal and distal epiphyses respectively. For the proximal epiphyseal and three diaphyseal measurement levels the mediolateral diameters were 8–13% greater than their respective craniocaudal diameters. Coursing distally from the tuberosities, diameters tapered in both planes towards the diaphysis. Along the diaphysis both planes marginally tapered distally, with 0.63 mm craniocaudal and 0.76 mm mediolateral variation between the proximal and distal third measurement levels. At the flattened distal epiphysis between epicondyles the craniocaudal external diameter was smallest at 15% of the mediolateral diameter.

**Table 1:** Micro-CT measurements of external diameter in both the craniocaudal (CC) and mediolateral (ML) planes of the koala humerus at each of the five measurement levels represented as mean with 95% confidence intervals.

Level	Direction	Measurement (mm)
Between tuberosities	CC	21.37 (19.55–23.19)
	ML	23.91 (21.09–26.73)
Proximal third	CC	10.80 (9.93–11.67)
	ML	12.42 (11.80–13.04)
Deltoid ridge	CC	10.79 (10.32–11.26)
	ML	11.79 (11.04–12.54)
Distal third	CC	10.17 (9.69–10.65)
	ML	11.66 (10.61–12.71)
Between epicondyles	CC	5.62 (4.97–6.27)
	ML	38.14 (36.76–39.52)

As the deltoid ridge ran from craniolateral to caudomedial the diameter measurement of the ridge was not included in the external diameter measurements. The craniolateral to caudomedial external diameter of the deltoid ridge measurement level was on average 18.33 mm (95% CI 17.46–19.2 mm).

### Internal diameter of bone

Humeral internal diameters were reported at all five measurement levels (Table 2). Internal diameter measurements along the entire bone were greater in the mediolateral plane than the craniocaudal plane. Craniocaudal internal diameter was greatest between tuberosities while mediolateral diameter was greatest between the epicondyles. The craniocaudal internal diameter between the tuberosities was on average 10% less than the mediolateral measurement. Along the diaphysis both craniocaudal and mediolateral internal diameters tapered distally. Craniocaudal internal diameters were 7% less than mediolateral measurements at the deltoid ridge, and 16% less at the proximal and distal third levels. Between the proximal and distal third measurement levels craniocaudal and mediolateral internal diameter means differed by 1.62 mm and 1.88 mm respectively. At the flattened distal epiphysis between epicondyles the craniocaudal internal diameter was smallest at 12% of the mediolateral measurement.

**Table 2:** Micro-CT measurements of internal diameter in both the craniocaudal (CC) and mediolateral (ML) planes of the koala humerus at each of the five measurement levels represented as mean with 95% confidence intervals.

Level	Direction	Measurement (mm)
Between tuberosities	CC	20.65 (18.91–22.39)
	ML	22.73 (20.11–25.35)
Proximal third	CC	8.18 (7.10–9.26)
	ML	9.49 (8.27–10.65)
Deltoid ridge	CC	7.72 (6.58–8.86)
	ML	8.24 (6.84–9.64)
Distal third	CC	6.56 (5.10–8.02)
	ML	7.61 (5.83–9.39)
Between epicondyles	CC	4.36 (3.74–4.98)
	ML	36.94 (35.33–38.55)

The medullary cavity was largely ovoid in shape through the diaphysis with a craniolateral projection into the deltoid ridge. The craniolateral to caudomedial internal diameter of the deltoid ridge measurement level was on average 16.41 mm (95% CI 15.47–17.35 mm).

### Cortical thickness

Humeral cortical thickness was reported at each measurement level (Table 3). Medial and caudal cortices were thicker than lateral and cranial cortices at the tuberosities, proximal third and deltoid ridge measurement levels. At the distal third and between the epicondyles the medial and lateral cortices were greater than the cranial and caudal cortices. The medial cortex was consistently thickest along the length of the entire bone. From proximal to distal along the length of the diaphysis all four cortices were a relatively homogenous thickness, with a very marginal increase progressing distally. Cortices were smaller in all four directions between tuberosities and epicondyles compared to diaphyseal thicknesses, with the thinnest cortices being between tuberosities.

**Table 3:** Micro-CT cortical thickness measurements in the cranial (Cr), caudal (Ca), medial (Me) and lateral (La) directions of the koala humerus at the five measurement levels represented as mean with 95% confidence intervals.

Level	Direction	Measurement (mm)
Between tuberosities	Cr	0.23 (0.17–0.29)
	Ca	0.33 (0.16–0.50)
	Me	0.74 (0.35–1.13)
	La	0.22 (0.05–0.39)
Proximal third	Cr	1.19 (0.76–1.62)
	Ca	1.43 (0.98–1.88)
	Me	1.48 (1.04–1.92)
	La	1.37 (1.01–1.73)
Deltoid ridge	Cr	1.52 (1.02–2.02)
	Ca	1.61 (1.22–2.00)
	Me	1.90 (1.07–2.73)
	La	1.61 (1.14–2.08)
Distal third	Cr	1.93 (1.35–2.51)
	Ca	1.72 (1.18–2.60)
	Me	2.01 (1.40–2.62)
	La	1.94 (1.10–2.78)
Between epicondyles	Cr	0.51 (0.33–0.69)
	Ca	0.51 (0.36–0.66)
	Me	0.87 (0.62–1.12)
	La	0.56 (0.43–0.69)

### Distal cancellous bone

The distal medullary cavity was 50% filled with cancellous bone at a mean of 84.55 mm (95% CI 76.45–92.65 mm) or 74% the length of bone, while cancellous bone filled 100% of the medullary cavity distally at a mean of 90.56 mm (95% CI 84.28–96.84 mm) or 79% the length of bone.

## Discussion

Lengths, internal and external diameters, cortical thicknesses, distal proportions of cancellous bone and other useful features were all documented in a group of skeletally mature koalas. Although CT scans were previously employed for morphometric analysis of the koala nasal cavity^10^, detailed appendicular skeletal morphometry has remained undocumented. Using micro-CT in this study provided much smaller slice capabilities for reconstructed images, allowing for more precise descriptive analysis aimed to benefit future clinical applications.

The medullary cavity of the diaphysis tapers distally, and throughout the entire bone the dimensions of the cavity are greater in the mediolateral plane. This is important for orthopaedic research as failure rates of implants such as the interlocking nail are dependent on their design and size^24^. In mature koalas, should implant stabilisation be implicated in fracture repair, the size and shape of the implant should cater for the tapering internal diameter to optimise stability and promote healing. The humeral medullary cavity also allows implants to have a marginally greater mediolateral than craniocaudal diameter. Intramedullary implants such as the interlocking nail are typically directed normograde through the medullary cavity, embedding into the distal cancellous bone for stability^25^. This study showed the distal cancellous bone fills the cavity approximately 79% down the length of bone. This will guide surgeons when developing surgical implant length recommendations for future fracture repairs. Other important details regarding the shape of the koala humerus that must be considered when developing orthopaedic techniques is the craniocaudal flattening of the epiphysis and the caudal curvature of the distal humerus. These factors may complicate how conventional implants embed into the distal cancellous bone. The flattening suggests the further distal the implant, the less craniocaudal space there will be to accommodate the implant, and the curvature of the bone suggests that anatomically pre-contoured implants might be required.

Diaphyseal cortical proportions were relatively homogenous with a very marginal increase in the thickness of more distal bone. From an orthopaedic perspective, this result suggests the diaphyseal cortex should have minimal variation in strength throughout its length^11^. Although slight, the increase in distal diaphyseal thickness may suggest the distal diaphysis can withstand slightly higher forces than proximal bone. However, it is important to note that bone strength does not only depend on cortical thickness, but also other structural factors (bone diameter and shape) and material properties of the bone (density and porosity)^26^. Cortical thickness and bone shape likely reflect the origin and insertion of surrounding soft tissue attachments. Regions of cortical and cancellous bone have been shown to remodel following dynamic physiological stress and external forces acting on the bone^27–29^. No distinct osseous features course medially as the deltoid and supinator ridges do laterally. The increased surface area provided by the deltoid ridge means that dynamic tensile forces associated with the pectoralis major and deltoid insertion sites^8^ can be distributed over a greater area requiring less focussed cortical strength. Similarly, at the distal diaphysis the supinator ridge marks the origins of the extensor carpi radialis longus and supinator longus muscles^8^. The medial surface has no bone protuberances and a comparatively thicker cortex. The thicker medial cortex could be useful for screw placement, as thicker cortices have greater screw loading strength^25,30^. The relatively uniform surface may also be ideal for minimally invasive osteosynthesis, should plate fixation be indicated^25^. Knowledge of how surrounding soft tissue structures relate to cortical thickness can help develop optimal surgical approaches to the koala humerus based on muscular attachment sites and bone strength.

This descriptive study on koala humeral morphometry has several limitations that need to be considered. Measurement parameters calculated in this study were not paired with koala sex, weight, or age. Analysis between cohorts defined by these variables may provide useful points of comparison when developing surgical recommendations. For example, male koala body size and weight is typically greater than females^31,32^. It is therefore possible that male humeral parameters could be larger than female measurements. Additionally, the dataset analysed was limited to a cohort of six mature, cadaveric koalas and may not be representative of cohorts that include geriatric or juvenile individuals. Finally, no statistical analysis was used in this descriptive study. Given the absence of previous studies, the small sample size and lack of a comparison group, statistical analysis would not provide any additional information.

Radiographic inspection of each humerus prior to inclusion in the study meant any immature or skeletally diseased animals were excluded. Therefore, this study provides useful anatomical details for orthopaedic researchers investigating treatment of humeral fractures in mature koalas. Despite this, individual koala reproductive and nutritional status remained unknown. These factors are known to influence bone development, quality and cancellous bone volume^11,31^. Being from an unmonitored wild population establishing these details can be inconsistent and is a limitation of this study.

Only right humeri were used in this study, so potential variation between left and right sides should be considered. Preferential limb use in koalas is unknown but has been demonstrated in other marsupial species including the brushtail possum^33^, and red-necked wallaby^34,35^. If there is continual preference for a particular limb, bone size and quality may change in response to additional dynamic force remodelling^27,28^. Studies have investigated koala muscle mass proportions between hind and forelimbs but not between individual limbs^5^. If preferential limb treatment is present in koalas it is unknown whether the effect would be substantial enough to make a difference to healthy koala bone morphometry.

Fracture location has been shown to be prognostic in influencing whether koalas can be successfully rehabilitated and released^2^. However, no previous studies have described the frequency or distribution of fracture types in specific koala bones. This may be the subject of future research as different repair techniques may be indicated based on the nature, location and characteristic of the presenting fracture^36^. Different fracture locations along the humerus provide unique biomechanical considerations that may influence the treatment type.

## Conclusion

This morphometric study described lengths, internal and external diameters, cortical thickness along with qualitative anatomical descriptions of the koala humerus. The detailed anatomical descriptions will provide a useful reference for future orthopaedic research exploring optimal humeral fracture repair techniques including implant size and type. New clinical applications can be developed to improve prognosis of injury and better facilitate successful rehabilitation and release of injured koalas.

## Methods

### Cadaveric samples

Six koala cadavers were obtained from the University of Sydney Veterinary Pathology Diagnostic Services and the University Veterinary Teaching Hospital Camden. No koala was euthanised purely for use in this study. The sample size included two male and four female koalas (n = 6) aged approximately 3–8 years old between 6.2 and 8.8 kg. The right forelimb from each koala cadaver was harvested from the trunk of the body and stored frozen at − 20 °C. Prior to radiography and micro-CT imaging, each limb was thawed at room temperature for 24 h.

### Imaging protocol

Radiographs of all humeri were conducted and reviewed by a board certified surgical specialist to confirm skeletal maturity and absence of skeletal disease before performing micro-CT. All humeri were positioned for standard orthogonal radiographs showing craniocaudal and mediolateral perspectives.

Micro-CT scans were performed at the Sydney Imaging Core Research Facility. Each humerus was scanned using “Total Body Accurate” (TB_A) settings with two CT bed positions to scan the entire length of bone. The CT scan parameters were 55kVp, 0.19 mAs and 75 ms for six minutes each, and these were reconstructed at 50 µm voxel size. The reconstructed NII files were saved and used for image analysis.

### Image analysis and data collection

Image analysis of the micro-CT images was performed using VivoQuant (version 4.0). Each bone was measured separately using two-dimensional images generated by multiplanar reconstruction (MPR) of the NII micro-CT scan files. All parameters were measured in triplicates from which a mean value was calculated and reported.

**Figure 2:**
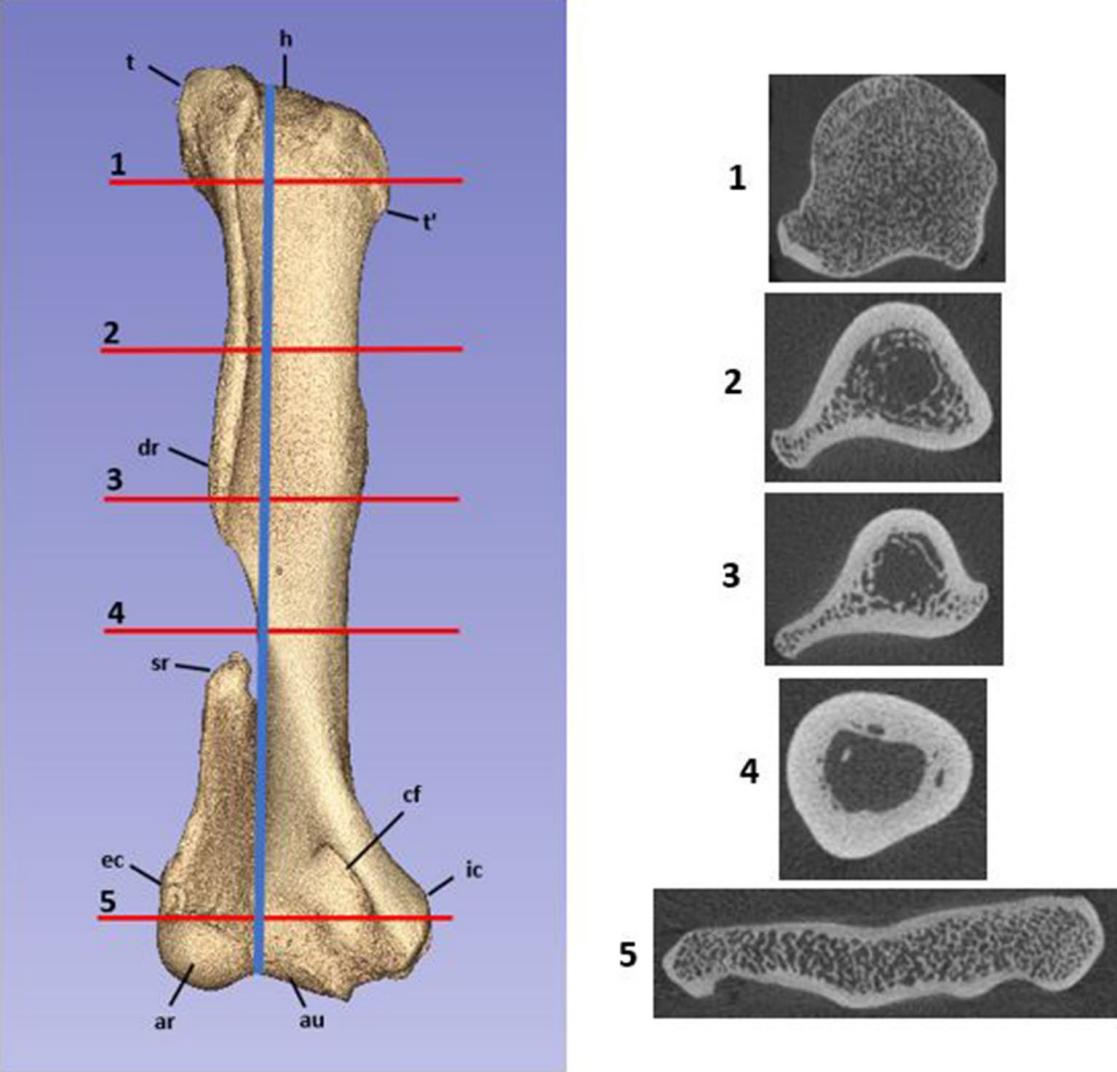
Cranial view of the koala humerus produced by 3D reconstruction of the micro-CT scan using Slicer version 4.10.2. Left of the image is lateral. Blue line indicates humeral length. Red lines indicate each measurement level of the humerus. 1 = between tuberosities; 2 = proximal third; 3 = deltoid ridge; 4 = distal third; 5 = between epicondyles. Identifying landmarks are also identified. Where h = humeral head; t = greater tuberosity; t’ = lesser tuberosity; dr = deltoid ridge; sr = supinator ridge; cf = supracondylar foramen; ec = external epicondyle; ic = internal epicondyle; ar = articular surface (radius); au = articular surface (ulna). Also included are the transverse slices of each measurement level with lateral on the left and cranial at the bottom of each image.

The length of the humerus was measured from the most proximal point of the humeral head to the base of the groove between the radial and ulnar articular surfaces at the distal epiphysis. Five transverse levels of bone were then determined for data collection (Fig. 2). The humeral diaphysis was divided into three levels, the proximal third, distal third and the deltoid ridge. Proximal and distal third levels were calculated as one and two thirds of the calculated humeral length respectively. The middle measurement was determined to be located at the level of the deltoid ridge, where there was the greatest bone protuberance diameter in the transverse plane. Two other measurement sites were located at the proximal and distal epiphyses. The proximal epiphyseal measurement was determined to be the site between greater and lesser tuberosities with the greatest diameter proximal to the humeral diaphysis. At the distal epiphysis, the measurement was taken at the widest section of bone midway between the medial and lateral epicondyles.

**Figure 3:**
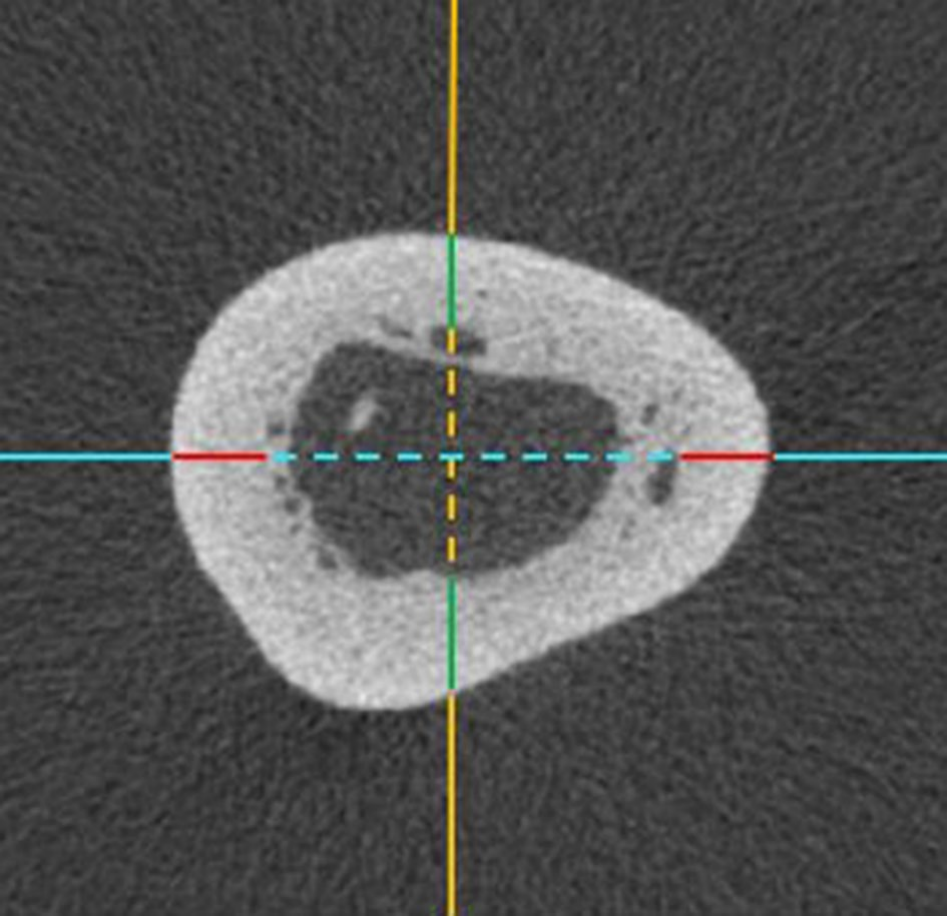
Transverse micro-CT slice taken from the distal third measurement site. Top of the image is cranial, left of the image is lateral. Solid orange = craniocaudal plane; solid blue = mediolateral plane; solid green = cranial and caudal cortical thicknesses; solid red = medial and lateral cortical thicknesses; dotted orange = internal diameter of craniocaudal plane; dotted blue = internal diameter of mediolateral plane.

For each humerus, transverse sections at each respective level described above were orientated and marked to determine the relative cranial, caudal, medial and lateral aspects of the bone. Internal and external diameters were measured at each level using the craniocaudal and mediolateral axes. Cortical bone thickness was measured as the width of cortical bone at each of the cranial, caudal, medial, and lateral aspects in a single transverse slice. (Fig. 3). The proportion of bone length distal to the medullary cavity filled with cancellous bone was also quantified. The distal cancellous bone length was measured as the distance along the length of the humerus, from humeral head origin, where cancellous bone comprised 50% and 100% of the medullary cavity. Qualitative descriptions of any unique or prominent bone features deemed to be potentially relevant to fracture repair, surgical implantation or bone healing in koalas were also documented.

### Ethics approval and consent to participate

The animals used had died, or were euthanized for reasons unrelated to the study and acquired in accordance with guideline GL001 from the University of Sydney animal ethics committee.

## Data Availability

The datasets used and/or analysed during the current study are available from the corresponding author on reasonable request.
